# Investigations of the Density and Solubility of Ammonium Perrhenate and Potassium Perrhenate Aqueous Solutions

**DOI:** 10.3390/ma16155481

**Published:** 2023-08-05

**Authors:** Szymon Orda, Michał Drzazga, Katarzyna Leszczyńska-Sejda, Mateusz Ciszewski, Alicja Kocur, Pola Branecka, Kacper Gall, Mateusz Słaboń, Marcin Lemanowicz

**Affiliations:** 1 Łukasiewicz Research Network—Institute of Non-Ferrous Metals, Centre of Hydroelectrometallurgy, ul. Sowińskiego 5, 44-100 Gliwice, Poland; michal.drzazga@imn.lukasiewicz.gov.pl (M.D.); katarzyna.leszczynska-sejda@imn.lukasiewicz.gov.pl (K.L.-S.); mateusz.ciszewski@imn.lukasiewicz.gov.pl (M.C.); 2 Department of Chemical Engineering and Process Design, Faculty of Chemistry, Silesian University of Technology, ul. ks. M. Strzody 7, 44-100 Gliwice, Poland; alicja.kocur@polsl.pl (A.K.); kacper.gall@student.polsl.pl (K.G.); mateusz.slabon@student.polsl.pl (M.S.); marcin.lemanowicz@polsl.pl (M.L.)

**Keywords:** rhenium, ammonium perrhenate, potassium perrhenate, densimetric method, solubility curve

## Abstract

Rhenium is largely used as an additive to nickel- and cobalt-based superalloys. Their resistance to temperature and corrosion makes them suitable for the production of turbines in civil and military aviation, safety valves in drilling platforms, and tools working at temperatures exceeding 1000 °C. The purity of commercial rhenium salts is highly important. Potassium, which is a particularly undesirable element, can be removed by recrystallization. Therefore, it is crucial to possess detailed knowledge concerning process parameters including the dissolved solid concentration and the resulting saturation temperature. This can be achieved using simple densimetric methods. Due to the fact that data concerning the physicochemical properties of ammonium perrhenate (APR) NH_4_ReO_4_ and potassium perrhenate (PPR) KReO_4_ are imprecise or unavailable in the scientific literature, the goal of this study is to present experimental data including the solubility and density of water solutions of both salts. In the experiments, a densimeter with a vibrating cell was used to precisely determine the densities. Although the investigated solutions did not fit into the earlier proposed mathematical model, some crucial conclusions could still be made based on the results.

## 1. Introduction

Metallic rhenium is a valuable metal characterized by parameters such as a density of 21.02 g/cm^−3^, a melting point of 3181 °C, a heat capacity of 0.137 J∙g^−1^∙K^−1^, a Young’s modulus of 463 GPa, and a hardness (Mohs scale) of 7.0 [1,2,3]. The application of rhenium includes different branches of industry: aviation, defense, petrochemicals, medicine and electricity. An average of 83% of rhenium is used for the production of superalloys [2,4,5]. The addition of metallic Re to nickel-based alloys increases thermal and corrosion resistance and thus the durability of materials used for jet engines [6]. The second area of application is the petrochemical industry. About 12% of rhenium is used in reforming high-octane gasoline [2,5]. Bi-metallic catalysts (Re-Pt) are characterized by significantly higher operation stability at high temperatures and under low pressure [7,8]. The lifetime of Re-Pt catalysts during the deposition of coal on surfaces is longer compared to traditional ones [7]. In medical applications, rhenium is used for the production of stents and X-ray lamps. Metal is also used in therapeutic medicine [5]. Radioactive isotope rhenium ^187^Re decays, emitting radiation, which is not dangerous to the human body [3,9]. Other applications of this metal include the production of thermocouples, heating elements, electrodes, electric contact points, flashbulbs, and electromagnets [4,10,11]. The development of the aerospace industry has had an influence on rhenium demand and thus metal prices.

Molybdenite concentrates from copper mines are the most important primary sources of rhenium [1,6,10,12,13]. Rhenium occurs there as ReS_2_. During the roasting of molybdenite concentrates, fumes and dust containing the volatile form of rhenium, Re_2_O_7_, are generated [14]. Fumes containing rhenium heptoxide in contact with water create scrubber liquor containing ReO_4_^−^. Then, after concentration, the ammonium perrhenate, the main commercial rhenium compound, is produced. Limited access to primary sources leads to the recycling of rhenium from spent catalysts and superalloy waste [15,16,17]. Nowadays, there exist numerous technologies including recovery for the rhenium from deactivated catalysts [7,8,18]. 

Ammonium perrhenate is the main commercial rhenium compound. The most important step in the production of its crystalline form is the separation of rhenium from solutions created during technological processes. The reported methods of rhenium separation involve ion exchange, adsorption, solvent extraction, and precipitation. In the first case, different types of resins have been investigated [4,6,10,12,19,20,21,22,23,24,25,26,27,28,29]. Rhenium from scrubber liquor may be adsorbed using active carbon or nano particles of iron(III) oxide coated with active carbon [30,31]. In the case of solutions enriched with rhenium a suitable approach is solvent extraction for which many different extractants have been studied [32,33,34,35]. Finally, apart from the above-mentioned methods, another form of rhenium recovery is precipitation [36,37]. 

Crude ammonium perrhenate (CAP) usually requires additional treatment, i.e., purification. One of the most undesirable pollutants is potassium. Potassium ions form a sparingly soluble compound—potassium perrhenate (PPR). Researchers have tested electrodialysis membranes and ion exchange to remove potassium from CAP [15,38,39,40]. However, CAP recrystallization easily removes impurities such as potassium. One can find just a few papers dedicated to the investigations of APR crystallization. There is one publication about the multistage recrystallization of CAP to remove potassium [41]. Apart from that, the influence of stirring on the recrystallization of ammonium perrhenate has been examined [42]. Other properties, the thermal expansion coefficient, the standard enthalpies formation, and the heat capacity single crystals APR and PPR, have also been determined [43,44,45,46]. It was found that the thermal expansion coefficient of ammonium perrhenate indicates an anomaly compared to potassium perrhenate [47]. The mutual solubility of sodium perrhenate in a water–ethanol system was also tested [48]. 

In order to properly carry out the crystallization process, it is obligatory to know the mechanism and kinetics of the APR and PPR crystallization process [49]. Due to limited or obsolete data concerning the physicochemical properties of APR and PPR water solutions, we decided to deeply investigate the solubilities and densities of these salts. Potassium perrhenate is the main impurity of ammonium perrhenate—the main commercial rhenium compound used for the production of metallic rhenium. This salt crystalizes from a ternary solution and since its solubility is significantly lower than the solubility of the main product, a recrystallization process has to be employed. Moreover, the performed research allowed us to make an attempt to fit the experimental data to the mathematical models available in the literature. In the future, this work will be a solid foundation for investigations into APR and PPR crystallization, like the process design and control or its numerical simulations.

## 2. Materials and Methods

Two rhenium salts were used for density measurements: ammonium perrhenate (analytical grade, INNOVATOR sp. z.o.o., Gliwice, Poland) and potassium perrhenate. The potassium salt was prepared via the neutralization of perrhenic acid by potassium hydroxide (analytical grade, Chempur, Piekary Slaskie, Poland). Perrhenic acid was obtained from the APR by the ion exchange method [50]. The saturated solutions of each salt were prepared using reverse osmosis (RO) water (0.06 μS cm^−1^ conductivity, Hydrolab, Straszyn, Poland). For both reagents’ phase compositions, determination was performed by X-ray diffraction. XRD analysis was performed using a Rigaku MiniFlex600 (Rigaku Co., Tokyo, Japan) diffractometer. The obtained diffractograms are included as Supplementary Materials (Figures S1 and S2).

Excess amounts of salt were dissolved in RO water in a thermostated vessel reactor of 0.5 × 10^−3^ m^3^. The saturated solution was stirred for 2 h using a magnetic stirrer at a constant temperature. The temperature was controlled by a thermostat Huber Ministats CC-K6 (temperature accuracy +/−0.02 K). After stabilization, a sample of saturated solution was collected by a syringe equipped with a microporous filter (0.22 μm pore size). To prevent crystallization during sampling, the syringe, filter, and needle were heated to 10 K above the saturation temperature each time before use. A small portion of solution (approximately 3 mL) was taken from the vessel and transferred to digital densimeter DMA 4500M (Anton Paar, Singapore), which determined the density with an accuracy of +/−5 × 10^−5^ g∙cm^−3^ at atmospheric pressure. The temperature was controlled automatically by the densimeter within ±0.02 K accuracy. The densities of the samples were determined based on the oscillating U-tube principle. The glass cell was excited and oscillated at a given frequency depending on the mass of the investigated fluid. The density was calculated based on the corresponding frequency. In contrast with classical methods, the precise measurements may be completed using small volumes of fluids. After 30 min, another measurement was made. If the two subsequently measured densities were similar, then it was assumed that the equilibrium state was achieved. The saturated solutions of APR and PPR salts were prepared in the same way.

The samples for the solubility and density measurements were collected from saturated solutions. The density was measured in the range between the saturation temperature and a 10 K higher temperature with 1 K steps. This process was performed automatically by the densimeter. Moreover, an additional sample was taken in order to determine the solubility of the salts using the gravimetric method. Multiple beakers were filled with samples and placed in a laboratory dryer at 105 °C. They were dried until a constant mass was achieved. The solubility was calculated on the basis of the mass difference. Experiments were carried out at six saturation temperatures: 283.15 K; 293.15 K; 303.15 K; 313.15 K; 323.15 K; and 333.15 K. All measurements were repeated three times.

## 3. Results and Discussion

### 3.1. Solubility Curve

The solubility data are presented in Tables S1 and S2, attached as Supplementary Materials. The experimental data can be described by the following mathematical model [51]:

(1) $$\log_{10}S=A+\frac{B}{T}+C\cdot \log_{10}T$$

Based on the experimental data coefficients, *A*, *B*, and *C* in Equation (1) were obtained using the least squares method (LSM). The values are presented in Table 1.

**Figure 1:**
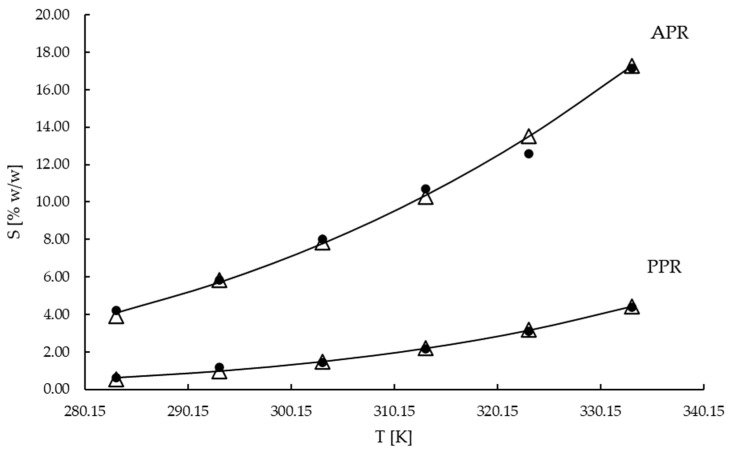
Solubility of NH_4_ReO_4_ and KReO_4_: ∆ experimental; ▬ calculated; ● literature [52].

Figure 1 presents the experimental data, the calculated values, and the data taken from the literature for ammonium and potassium perrhenate. The solubilities of the two salts were compared.

The standard deviation bars are not presented in the figure due to their small values. The highest standard deviation was equal to 0.33 % *w*/*w*. As one may notice, the points obtained on the basis of our experiments are in better agreement with the mathematical model than the data available in the literature. The solubility of ammonium perrhenate changes noticeably with temperature, which would seem to make the isohydric crystallization by cooling a suitable process for its production. However, Figure 1 perfectly illustrates the challenge concerning the removal of potassium perrhenate. As one may notice, the solubility of this salt is very low compared with ammonium perrhenate. Only at 60 °C does the solubility of KReO_4_ match the solubility of NH_4_ReO_4_ at 10 °C.

### 3.2. Density Mesurements

#### 3.2.1. Ammonium Perrhenate

**Figure 2:**
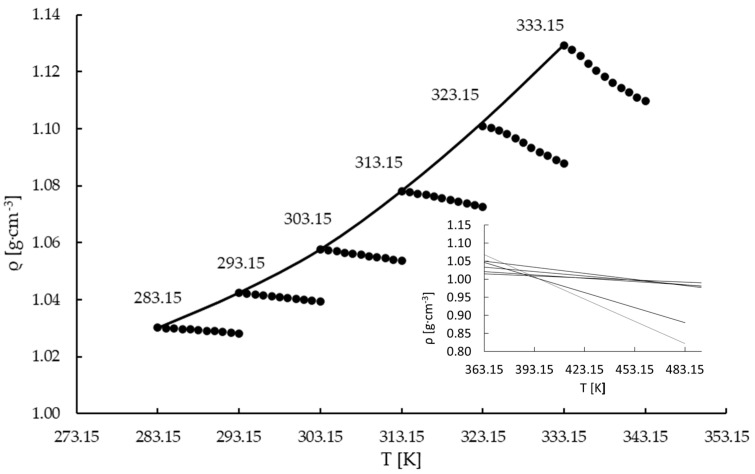
Density vs. temperature of concentrated NH_4_ReO_4_ solutions (●) and density curve in temperature saturation (▬).

Experimental data on the density measurements for the undersaturated region are presented in Table S3 in the Supplementary Materials. For the average values of density, standard deviations were calculated, and they are presented in the Supplementary Materials. Based on the collected data, Figure 2 was plotted. In the limited range, close to the saturation line, the density of the solution can be described by the linear function of temperature [53,54]:

(2) ρ=a·T+b

The linear equation coefficients (*a* and *b*) and the coefficient of determination (R^2^) for the undersaturated region are given in Table 2 for all investigated saturation temperatures. The same type of linear correlation was obtained by Frej et al. [53], Marciniak [54,55], and Bogacz et al. [55,56]. 

The density curve at the saturation temperature (Figure 2) is described by the following second-order polynomial equation:

(3) $$\rho =a\cdot T^{2}+b\cdot T+c$$

In Equation (3), the correlations coefficients (*a*, *b*, and *c*) could be obtained by using the LSM and the experimental density data. In Table 3, the coefficients and determination coefficients are presented.

It can be observed that the correlation slope decreases with the increase in the saturation temperature. Using the slope coefficients of Equation (2) from Table 2 for the extrapolated temperature range, an attempt was made to determine a construction point (Figure 2). It can be seen that for temperatures 323.15 and 333.15 K, the *a* coefficient is significantly lower than for lower temperatures. Thus, the extrapolated density lines did not intersect at the same point, and determination of construction points for the investigated temperature range was not possible. 

**Figure 3:**
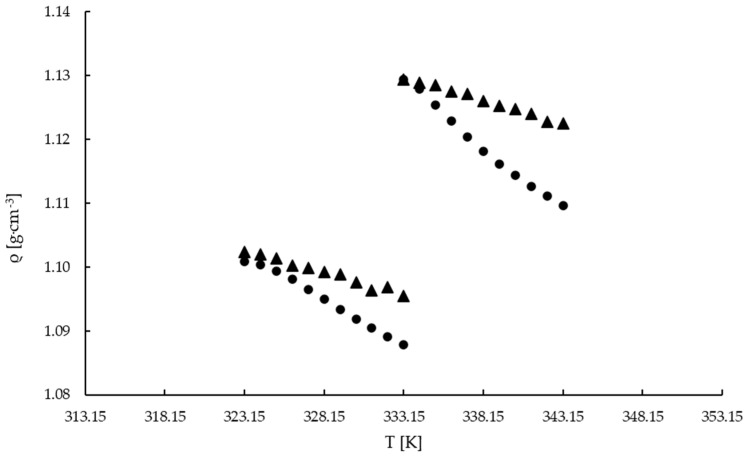
Density measurements of NH_4_ReO_4_ solutions: ● automatic; ▲ manual.

Additional density measurements for ammonium perrhenate solutions at 323.15 K and 333.15 K in the unsaturated region were performed by measuring a fresh sample of saturated solution. The density data are presented in the Supplementary Materials in Table S3. For each measurement, a fresh sample of saturated solution was injected into the device, its temperature was adjusted to the target value, and the density was measured. Figure 3 presents the comparison of the density data for the solution saturation temperatures of 323.15 K and 333.15 K for automatic measurement (temperature scan of density—a whole data series was made based on one sample) and manual measurements (each point in the series was determined based on the fresh sample). It may be noticed that in the first case the slope coefficient is visibly higher compared to the second case. This is consistent with the data presented in Figure 3 where the trend of these two data series deviated from the rest of the samples. Also, the correlation coefficients for Equation (2) and the determination coefficients for the undersaturated regions were calculated, and they are presented in Table 4. In our opinion, this behavior may be explained by the formation of gas (ammonia) microbubbles during the measurements [53]. This observation is also supported by the thermodynamic calculations performed using HSC Chemistry 9.6.1 software (Outotec, module Gem—Equilibrium Composition), according to which ammonia evolution is possible for temperatures exceeding 320 K (Figure S3 in the Supplementary Materials). It is worth noting that the first points for both measurement methods are identical. This simply means that the properties of the sample changed during the measurement. The temperature scan took about 1 h. For such a long period of time and at elevated temperatures microbubbles of ammonia could be created within the sample. Since the density measurements are based on cell vibration analysis, the inertia of bubbles resulted in a decrease in the measured value. The longer the sample was measured, the higher the deviation that was achieved due to the higher accumulation of bubbles. 

Due to the relatively low solubility of the salt, its impact on the solution density was limited. Yet, the polynomial nature of the saturation line was preserved. These data are an interesting starting point for future investigations on crystallization and solubility kinetics. Moreover, the phenomenon of ammonium formation and its impact on multicomponent crystallization should be investigated more deeply in the future.

#### 3.2.2. Potassium Perrhenate

**Figure 4:**
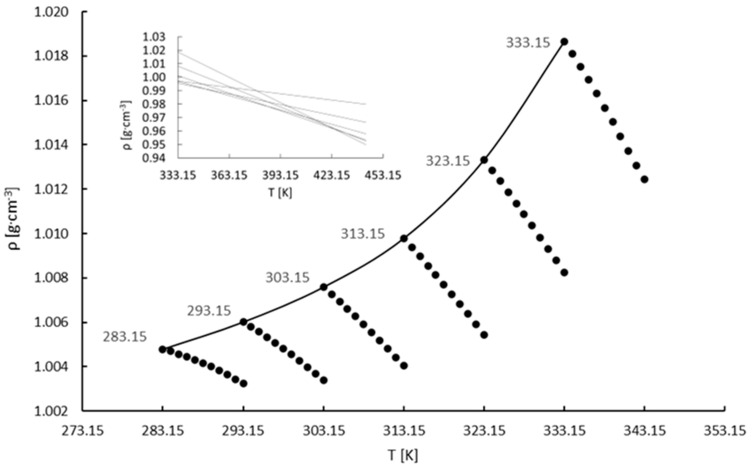
Density vs. temperature of the concentrated KReO_4_ solutions.

The density data on potassium perrhenate and the standard deviations are presented in the Supplementary Materials in Table S5. Based on the density values and the same procedure as for ammonium perrhenate, Figure 4 was plotted. The coefficients (a, b, and R^2^) describing the slope of the density lines are presented in Table 5. Moreover, the coefficients in Equation (3) for the potassium perrhenate solution are presented in Table 6.

**Figure 5:**
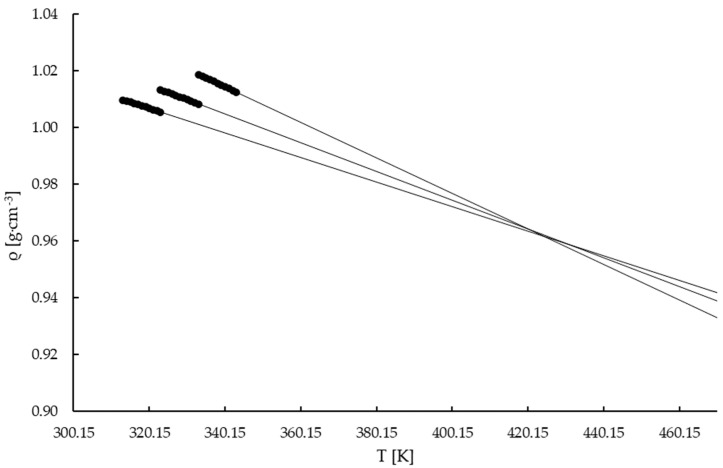
Construction point of the concentrated KReO_4_ solution.

Additional PPR density measurements using fresh solutions were not made. At low saturation temperatures, i.e., 283.15, 293.15, and 303.15 K, it was not possible to determine the construction point as the extrapolated lines did not intersect at a single point. It is shown in Figure 4. This may be related to the low solubility of PPR compared with APR (Figure 1). When the saturation temperatures decrease the change in density with temperature is lower. This can be observed in Figure 4. On the other hand, by using data for saturation temperatures of 313.15, 323.15, and 333.15 K, the construction point was obtained and plotted in Figure 5. All extrapolated lines intersect at one pole point, whose coordinates are (424.50 K, 0.96168 g∙cm^−3^) (Figure 5). Thus, the methodology for the determination of the construction point for potassium perrhenate solutions is appropriate at temperatures higher than 303.15K. It is important to emphasize that the obtained pole point has no physical meaning and it is used only for calculations [56].

In most cases, the application of the densimetric method to aqueous solutions of salts allows one to construct the pole point. Then, based on the density measurements, the pole point may be used for the determination of the saturation temperature [55,56]. In our opinion, the reason why the experimental data did not fit to the mathematical model proposed by Bogacz et al. [56] is associated with the solubility of the salts. Bogacz et al. investigated potassium chloride and potassium sulphate, whose solubilities significantly surpasses the solubilities of APR and PPR. It is also likely that the ions’ interaction plays a crucial role in the analysis of the behavior of such saturated systems.

As in the case of ammonium perrhenate, the presence of salt had a small impact on the densities of the solutions, which were slightly higher than for the base liquid, i.e., water. Due to the significant difference between the solubilities of both salts the densities of the ammonium perrhenate solutions were always higher than the solutions of potassium perrhenate. Once again, the second-order polynomial of the saturation curve was preserved. 

## 4. Conclusions

The densities and solubilities of aqueous solutions of APR and PPR were examined. In the case of solubility, the experimental data were correlated as a function of temperature using empirical equations, and good agreement was found (even better than when compared to the data already available in the literature). Based on the density data, an attempt was made to fit a mathematical model proposed in the literature. Unfortunately, it was impossible to find the pole points for both salts for the whole investigated temperature range. Surprisingly, it was noticed that for the ammonia salt, microbubbles were created during the density measurements for higher temperatures, which had a significant impact on the readings. For these cases, manual measurements had to be performed. The reason why the experimental data did not fit the mathematical model is, in our opinion, the low solubility of the investigated salts in relation to the systems for which the model was developed. Still, this work will be a solid foundation for investigations on APR and PPR crystallization, like the process design and control or its numerical simulation.

## Figures and Tables

**Table 1:** Regression and correlation coefficients of Equation (1).

Coefficient	Ammonium Perrhenate	Potassium Perrhenate
A	13.77561	13.99451
B	−1590.72975	−2009.10125
C	−3.07759	−2.90039
R^2^	0.99959	0.99951
A	13.77561	13.99451

**Table 2:** Coefficients and the determination coefficients for undersaturated NH_4_ReO_4_ solutions (automatic measurement). The second column represents the density of the saturated solution.

T	ρ	a	b	R^2^
[K]	[g∙cm^−3^]	[g∙cm^−3^∙K^−1^]	[g∙cm^−3^]	[-]
283.15	1.03009	−0.00019	1.08392	0.99320
293.15	1.04246	−0.00031	1.13205	0.99845
303.15	1.05765	−0.00041	1.18075	0.99933
313.15	1.07816	−0.00056	1.25512	0.99677
323.15	1.10233	−0.00139	1.55029	0.99369
333.15	1.12943	−0.00205	1.81105	0.99234

**Table 3:** Coefficients and determination coefficient of polynomial Equation (3) for NH_4_ReO_4_.

a × 10^5^	b	c	R^2^
1.85031	−0.00916	2.10943	0.98980

**Table 4:** Coefficients and the determination coefficients for undersaturated NH_4_ReO_4_ solutions (manual measurements).

T	ρ	a	b	R^2^
[K]	[g∙cm^−3^]	[g∙cm^−3^∙K^−1^]	[g∙cm^−3^]	[-]
323.15	1.02334	-0.00069	1.32514	0.98140
333.15	1.12943	-0.00073	1.37276	0.99253

**Table 5:** Coefficients and the square of the Pearson correlation coefficients for undersaturated KReO_4_ solutions.

T	Ρ	a	b	R^2^
[K]	[g∙cm^−3^]	[g∙cm^−3^∙K^−1^]	[g∙cm^−3^]	[-]
283.15	1.00479	−0.00016	1.04893	0.98903
293.15	1.00601	−0.00026	1.08350	0.99751
303.15	1.00759	−0.00035	1.11526	0.99908
313.15	1.00978	−0.00043	1.14579	0.99949
323.15	1.01332	−0.00051	1.17722	0.99971
333.15	1.01865	−0.00063	1.22731	0.99939

**Table 6:** Coefficients and determination coefficient of polynomial Equation (3) for KReO_4_.

a × 10^6^	b	c	R^2^
3.93827	−0.00221	1.29264	0.96180

## Data Availability

Not applicable.

## Supplementary Materials

The following supporting information can be downloaded at: https://www.mdpi.com/article/10.3390/ma16155481/s1. Figure S1: XRD analysis of NH_4_ReO_4_; Figure S2: XRD analysis of KReO_4_; Figure S3: Equilibrium amount of ammonia evolved from saturated NH_4_ReO_4_ aqueous solution vs. temperature (HSC Chemistry 9.6.1, Gem module); Table S1: Solubility of ammonium perrhenate; Table S2: Solubility of potassium perrhenate; Table S3: APR density versus temperature (automatic measurements); Table S4: APR density versus temperature (manual measurements); Table S5: PPR density versus temperature.

## References

[1] Lunk H.-J., Drobot D.V., Hartl H. (2021). Discovery, Properties and Applications of Rhenium and Its Compounds. ChemTexts.

[2] Leszczyńska-Sejda K., Benke G., Kopyto D., Chmielarz A., Drzazga M., Ciszewski M., Kowalik P., Goc K., Bugla K., Grabowski T. Development of Rhenium Technologies in Poland. Proceedings of the Conference Paper Proceeding Congress European Metallurgical Conference EMCGDMB.

[3] Millensifer A.T., Sinclair D., Jonasson I., Lipmann A., Gunn G. (2013). Rhenium. Critical Metals Handbook.

[4] Lutskiy D.S., Ignatovich A.S., Sulimova M.A. (2019). Determination of the Sorption Characteristics of Ammonium Perrenate Ions on Anion Exchange Resin AV-17-8. J. Phys. Conf. Ser..

[5] (2019). Rhenium: Outlook to 2029.

[6] Zhang B., Liu H.-Z., Wang W., Gao Z.-G., Cao Y.-H. (2017). Recovery of Rhenium from Copper Leach Solutions Using Ion Exchange with Weak Base Resins. Hydrometallurgy.

[7] Kasikov L.G., Petrova A.M. (2009). Processing of Deactivated Platinum-Rhenium Catalysts. Theor. Found. Chem. Eng..

[8] Angelidis T.N., Rosopoulou D., Tzitzios V. (1999). Selective Rhenium Recovery from Spent Reforming Catalysts. Ind. Eng. Chem. Res..

[9] Leddicotte G.W. (1981). The Radiochemistry of Rhenium.

[10] Lan X., Liang S., Song Y. (2006). Recovery of Rhenium from Molybdenite Calcine by a Resin-in-Pulp Process. Hydrometallurgy.

[11] John D.A., Seal R.R., Polyak D.E. (2017). Critical Mineral Resources of the United States—Economic and Environmental Geology and Prospects for Future Supply.

[12] Maltseva E.E., Blokhin A.A., Murashkin Y.V., Mikhaylenko M.A. (2017). An Increase in Purity of Ammonium Perrhenate Solutions with Respect to Molybdenum(IV) with the Sorption Recovery of Rhenium(VII) from Mo-Containing Solutions. Russ. J. Non-Ferr. Met..

[13] Fang D., Song Z., Zhang S., Li J., Zang S. (2017). Solvent Extraction of Rhenium(VII) from Aqueous Solution Assisted by Hydrophobic Ionic Liquid. J. Chem. Eng. Data.

[14] Ammann P.R., Loose T.A. (1972). Rhenium Volatilization during Molybdenite Roasting. Metall. Mater. Trans. B.

[15] Guro V.P. Ammonium Perrhenate Purification and Rhenium Recovery from Heat-Resistant Rhenium-Nickel Superalloys. Proceedings of the 21st International Conference on Metallurgy and Materials.

[16] Srivastava R.R., Kim M., Lee J. (2016). Novel Aqueous Processing of the Reverted Turbine-Blade Superalloy for Rhenium Recovery. Ind. Eng. Chem. Res..

[17] Tang J., Sun Y., Hou G., Ding Y., He F., Zhou Y. (2018). Studies on Influencing Factors of Ammonium Rhenate Recovery from Waste Superalloy. Appl. Sci..

[18] Kennth N.H., Xinghui M. (1996). Recovery of Platinum Group Metals and Rhenium from Materials Using Halogen Reagents. U.S. Patent.

[19] Cyganowski P., Cierlik A., Leśniewicz A., Pohl P., Jermakowicz-Bartkowiak D. (2019). Separation of Re(VII) from Mo(VI) by Anion Exchange Resins Synthesized Using Microwave Heat. Hydrometallurgy.

[20] Fisher S.A., Meloche V.W. (1952). Ion Exchange Separation of Rhenium from Molybdenum. Anal. Chem..

[21] Guo X., Ma Z., Li D., Tian Q., Xu Z. (2019). Recovery of Re(VII) from Aqueous Solutions with Coated Impregnated Resins Containing Ionic Liquid Aliquat 336. Hydrometallurgy.

[22] Kholmogorov A.G., Kononova O.N., Kachin S.V., Ilyichev S.N., Kryuchkov V.V., Kalyakina O.P., Pashkov G.L. (1999). Ion Exchange Recovery and Concentration of Rhenium from Salt Solutions. Hydrometallurgy.

[23] Mozammel M., Sadrnezhaad S.K., Badami E., Ahmadi E. (2007). Breakthrough Curves for Adsorption and Elution of Rhenium in a Column Ion Exchange System. Hydrometallurgy.

[24] Nebeker N., Hiskey J.B. (2012). Recovery of Rhenium from Copper Leach Solution by Ion Exchange. Hydrometallurgy.

[25] Shu Z., Yang M. (2010). Adsorption of Rhenium(VII) with Anion Exchange Resin D318. Chin. J. Chem. Eng..

[26] Virolainen S., Laatikainen M., Sainio T. (2015). Ion Exchange Recovery of Rhenium from Industrially Relevant Sulfate Solutions: Single Column Separations and Modeling. Hydrometallurgy.

[27] Xiong C., Yao C., Wu X. (2008). Adsorption of Rhenium(VII) on 4-Amino-1,2,4-Triazole Resin. Hydrometallurgy.

[28] Zagorodnyaya A.N., Abisheva Z.S., Sharipova A.S., Sadykanova S.E., Bochevskaya Y.G., Atanova O.V. (2013). Sorption of Rhenium and Uranium by Strong Base Anion Exchange Resin from Solutions with Different Anion Compositions. Hydrometallurgy.

[29] Zakhar’yan S.V., Gedgagov E.I. (2013). Anion-Exchange Separation of Rhenium and Selenium in Schemes for Obtaining Ammonium Perrhenate. Theor. Found. Chem. Eng..

[30] Seo S., Choi W.S., Yang T.J., Kim M.J., Tran T. (2012). Recovery of Rhenium and Molybdenum from a Roaster Fume Scrubbing Liquor by Adsorption Using Activated Carbon. Hydrometallurgy.

[31] Vosough M., Shahtahmasebi N., Behdani M. (2016). Recovery Rhenium from Roasted Dust through Super Para-Magnetic Nano-Particles. Int. J. Refract. Met. Hard Mater..

[32] Xiong Y., Lou Z., Yue S., Song J., Shan W., Han G. (2010). Kinetics and Mechanism of Re(VII) Extraction with Mixtures of Tri-Alkylamine and Tri-n-Butylphosphate. Hydrometallurgy.

[33] Sato T., Sato K. (1990). Liquid-Liquid Extraction of Rhenium (VII) from Hydrochloric Acid Solutions by Neutral Organophosphorus Compounds and High Molecular Weight Amines. Hydrometallurgy.

[34] Ali M.C., Suzuki T., Tachibana Y., Sasaki Y., Ikeda Y. (2012). Selective Extraction of Perrhenate Anion in Nitric Acid Solution Using 2,2′-(Imino)Bis(N,N′-Dioctylacetamide) as an Extractant. Sep. Purif. Technol..

[35] Gerhardt N.I., Palant A.A., Dungan S.R. (2000). Extraction of Tungsten (VI), Molybdenum (VI) and Rhenium (VII) by Diisododecylamine. Hydrometallurgy.

[36] Melaven A.D., Bacon J.A. (1947). Process for Recovering Rhenium 1947. U.S. Patent.

[37] Zagorodnyaya A.N., Abisheva Z.S. (2002). Rhenium Recovery from Ammonia Solutions. Hydrometallurgy.

[38] Zagorodnyaya A.N., Abisheva Z.S., Agapova L.Y., Sharipova A.S. (2019). Purification of Crude Ammonium Perrhenate from Potassium by Recrystallization, Sorption, and Membrane Electrodialysis. Theor. Found. Chem. Eng..

[39] Zagorodnyaya A.N., Abisheva Z.S., Sadykanova S.E., Sharipova A.S., Akcil A. (2017). Purification of Ammonium Perrhenate Solutions from Potassium by Ion Exchange. Miner. Process. Extr. Metall. Rev..

[40] Leszczyńska-Sejda K., Majewski T., Benke G., Piętaszewski J., Anyszkiewicz K., Michałowski J., Chmielarz A. (2012). Production of High-Purity Ammonium Perrhenate for W–Re–Ni–Fe Heavy Alloys. J. Alloys Compd..

[41] Zagorodnyaya A.N., Sharipova A.S., Linnik K.A., Abisheva Z.S. (2018). Multi-Stage Recrystallization of Crude Ammonium Perrhenate. Theor. Found. Chem. Eng..

[42] Tang J., Feng L., Zhang C., Sun Y., Wang L., Zhou Y., Fang D., Liu Y. (2020). The Influences of Stirring on the Recrystallization of Ammonium Perrhenate. Appl. Sci..

[43] Kruger G.J., Reynhardt E.C. (1978). Ammonium Perrhenate at 295 and 135 K. Acta Crystallogr. Sect. B.

[44] Brown R.J.S., Smeltzer J.G., Heyding R.D. (1976). Nuclear Quadrupole Resonance and Thermal Expansion in Perrhenate Salts. J. Magn. Reson..

[45] Weir R.D., Staveley L.A.K. (1980). The Heat Capacity and Thermodynamic Properties of Potassium Perrhenate and Ammonium Perrhenate from 8 to 304 K. J. Chem. Phys..

[46] Reynolds E.M., Yu M., Thorogood G.J., Brand H.E.A., Poineau F., Kennedy B.J. (2019). Thermal Expansion of Ammonium Pertechnetate and Ammonium Perrhenate. J. Solid State Chem..

[47] Johnson R.A., Rogers M.T. (1974). Anomalous Temperature Dependence of the NQR Frequency in NH_4_ReO_4_. J. Magn. Reson..

[48] Casas J.M., Sepúlveda E., Bravo L., Cifuentes L. (2012). Crystallization of Sodium Perrhenate from NaReO_4_–H_2_O–C_2_H_5_OH Solutions at 298 K. Hydrometallurgy.

[49] Lemanowicz M., Mielańczyk A., Walica T., Kotek M., Gierczycki A. (2021). Application of Polymers as a Tool in Crystallization—A Review. Polymers.

[50] Leszczyńska-Sejda K., Benke G., Chmielarz A., Anyszkiewicz K. (2009). Methods of Synthesis of Perrhenic Acid from Aqueous Solutions of Ammonium Perrhenate. Hydrometallurgy.

[51] Carl L.Y. (2012). The Yaws Handbook of Physical Properties for Hydrocarbons and Chemicals.

[52] Struwe F., Pietsch E. (1972). Gmelins Handbuch der Anorganischen Chemie.

[53] Ghosh S.K. (1956). Decomposition of Ammonium Nitrite in Solution. Z. Phys. Chem..

[54] Marciniak B. (2012). Density and ultrasonic velocity of undersaturated and supersaturated solutions of fluoranthene in trichloroethylene, and study of their metastable zone width. J. Cryst. Growth..

[55] Bogacz W. (2017). Densimetric method for determination of potassium sulphate aqueous solutions saturation point Densymetryczna metoda wyznaczania punktu nasycenia wodnych roztworów siarczanu(VI) potasu. Przem. Chem..

[56] Bogacz W., Al-Rashed M.H., Lemanowicz M., Wójcik J. (2016). A Simple Densimetric Method to Determine Saturation Temperature of Aqueous Potassium Chloride Solution. J. Solut. Chem..

