# Supplementary material for: Investigations of the Density and Solubility of Ammonium Perrhenate and Potassium Perrhenate Aqueous Solutions
Source: Materials (Basel). 2023 Aug 5;16(15):5481. doi: 10.3390/ma16155481 (PMC10420235; doi:10.3390/ma16155481)
Supplement: Supplementary file 1 [file materials-16-05481-s001.zip › materials-2518588-supplementary.pdf]

Supplementary Materials

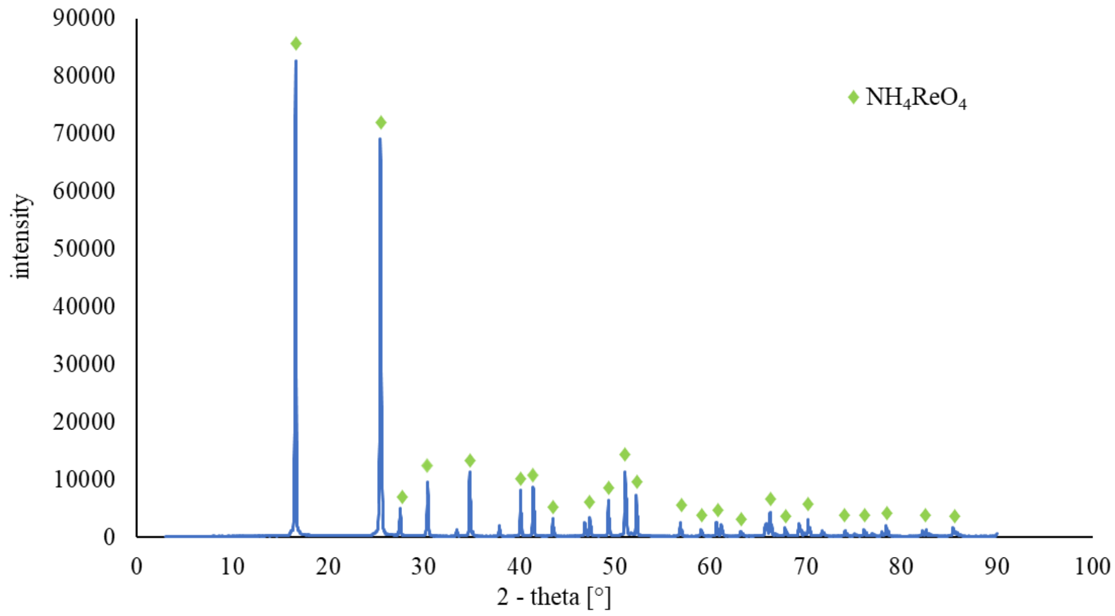

Figure S1. XRD analysis of NH_4ReO_4.

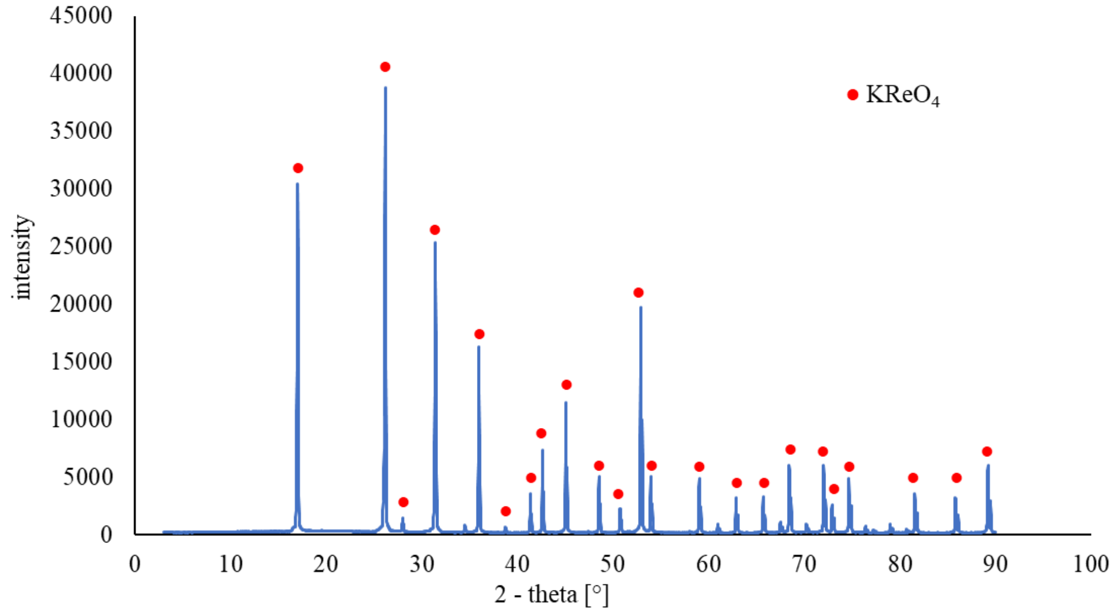

Figure S2. XRD analysis of $KReO_4$.

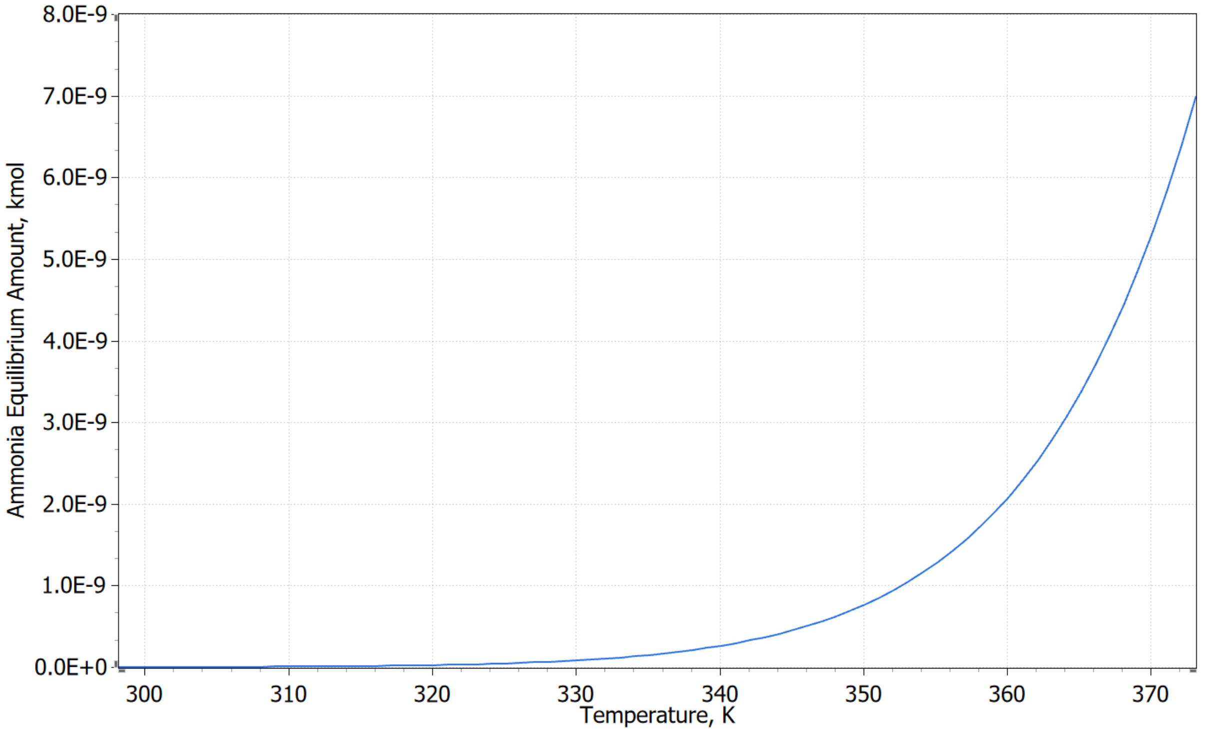

Figure S3. Equilibrium amount of ammonia evolved from saturated NH4ReO4 aqueous solution vs. temperature (HSC Chemistry 9.6.1, Gem module).

Table S1. Solubility of NH_4ReO_4.

Temperature [K]	Literature [52]		Experimental		STD	Calculated
	[g/100g H_2O]	[%w/w]	[g/100g H_2O]	[%w/w]	[%w/w]	[%w/w]
283.15	4.40	4.21	4.09	3.92	0.16	4.09
293.15	6.20	5.48	6.19	5.83	0.07	5.71
303.15	8.70	8.00	8.51	7.84	0.16	7.78
313.15	12.00	10.71	11.45	10.28	0.27	10.35
323.15	14.40	12.59	15.64	13.53	0.30	13.49
333.15	20.70	17.15	20.85	17.25	0.33	17.26

Table S2. Solubility of $KReO_4$.

Temperature [K]	Literature [52]		Experimental		STD	Calculated
	[g/100g H_2O]	[%w/w]	[g/100g H_2O]	[%w/w]	[%w/w]	[%w/w]
283.15	0.62	0.62	0.56	0.55	0.07	0.61
293.15	1.21	1.20	0.99	0.98	0.03	0.97
303.15	1.47	1.45	1.49	1.47	0.02	1.48
313.15	2.22	2.17	2.27	2.22	0.02	2.19
323.15	3.20	3.10	3.29	3.18	0.06	3.16
333.15	4.58	4.38	4.62	4.41	0.08	4.44

Table S3. NH_4ReO_4 density versus temperature (automatic measurements).

Saturation temperature [K]								
**283.15**			**293.15**			**303.15**		
**T [K]**	**ϱ [g·cm⁻³]**	**STD [g·cm⁻³] ·10⁴**	**T [K]**	**ϱ [g·cm⁻³]**	**STD [g·cm⁻³] ·10⁴**	**T [K]**	**ϱ [g·cm⁻³]**	**STD [g·cm⁻³] ·10⁴**
283.15	1.03009	3.98	293.15	1.04246	1.43	303.15	1.05765	9.19
284.15	1.02995	3.98	294.15	1.04220	1.43	304.15	1.05728	9.20
285.15	1.02980	3.98	295.15	1.04192	1.43	305.15	1.05690	9.22
286.15	1.02964	3.98	296.15	1.04163	1.40	306.15	1.05651	9.17
287.15	1.02947	3.94	297.15	1.04134	1.40	307.15	1.05612	9.15
288.15	1.02929	3.97	298.15	1.04104	1.40	308.15	1.05572	9.15
289.15	1.02909	3.94	299.15	1.04073	1.42	309.15	1.05531	9.15
290.15	1.02888	3.94	300.15	1.04041	1.40	310.15	1.05489	9.15
291.15	1.02866	3.93	301.15	1.04009	1.43	311.15	1.05446	9.10
292.15	1.02844	3.97	302.15	1.03975	1.43	312.15	1.05403	9.12
293.15	1.02820	3.97	303.15	1.03941	1.43	313.15	1.05359	9.10
313.15			323.15			333.15		
**T [K]**	**ϱ [g·cm⁻³]**	**STD [g·cm⁻³] ·10⁴**	**T [K]**	**ϱ [g·cm⁻³]**	**STD [g·cm⁻³] ·10⁴**	**T [K]**	**ϱ [g·cm⁻³]**	**STD [g·cm⁻³] ·10⁴**
313.15	1.07816	0.55	323.15	1.10092	2.30	333.15	1.12938	4.25
314.15	1.07768	0.60	324.15	1.10032	2.05	334.15	1.12786	12.55
315.15	1.07720	0.50	325.15	1.09938	2.60	335.15	1.12543	25.60
316.15	1.07670	0.30	326.15	1.09808	4.05	336.15	1.12291	30.75
317.15	1.07617	0.10	327.15	1.09652	6.40	337.15	1.12044	32.85
318.15	1.07562	0.65	328.15	1.09497	7.55	338.15	1.11814	33.25
319.15	1.07504	1.40	329.15	1.09331	9.65	339.15	1.11617	33.00
320.15	1.07444	2.25	330.15	1.09183	10.80	340.15	1.11433	32.00
321.15	1.07382	3.25	331.15	1.09043	11.65	341.15	1.11266	31.50
322.15	1.07317	4.30	332.15	1.08911	12.30	342.15	1.11111	31.65
323.15	1.07251	5.35	333.15	1.08786	12.80	343.15	1.10969	31.30

Table S4. NH_4ReO_4 density versus temperature (manual measurments).

Saturation temperature					
**323.15**			**333.15**		
**T [K]**	**ϱ [g·cm⁻³]**	**STD [g·cm⁻³] ·10⁴**	**T [K]**	**ϱ [g·cm⁻³]**	**STD [g·cm⁻³] ·10⁴**
323.15	1.10233	2.74	333.15	1.12943	3.61
324.15	1.10201	4.00	334.15	1.12895	5.34
325.15	1.10136	2.74	335.15	1.12855	9.31
326.15	1.10030	5.10	336.15	1.12757	122
327.15	1.09989	3.91	337.15	1.12712	8.70
328.15	1.09925	77.9	338.15	1.12601	5310
329.15	1.09882	94.2	339.15	1.12533	109
330.15	1.09763	5.33	340.15	1.12479	7.43
331.15	1.09642	5.11	341.15	1.12398	112
332.15	1.09685	2.64	342.15	1.12281	116
333.15	1.09554	2.30	343.15	1.12252	104

Table S5. $KReO_4$ density versus temperature.

Saturation temperature [K]								
**283.15**			**293.15**			**303.15**		
**T [K]**	**ϱ [g·cm^{-3}]**	**STD [g·cm^{-3}] ·10^5**	**T [K]**	**ϱ [g·cm^{-3}]**	**STD [g·cm^{-3}] ·10^5**	**T [K]**	**ϱ [g·cm^{-3}]**	**STD [g·cm^{-3}] ·10^5**
283.15	1.00479	3.27	293.15	1.00601	7.32	303.15	1.00759	9.88
284.15	1.00469	3.27	294.15	1.00579	7.32	304.15	1.00727	9.88
285.15	1.00457	3.68	295.15	1.00556	7.32	305.15	1.00694	9.88
286.15	1.00445	3.27	296.15	1.00532	7.32	306.15	1.00660	10.20
287.15	1.00431	3.27	297.15	1.00507	7.32	307.15	1.00626	9.88
288.15	1.00416	3.27	298.15	1.00481	7.32	308.15	1.00591	9.42
289.15	1.00400	3.3	299.15	1.00454	7.32	309.15	1.00555	9.42
290.15	1.00383	3.3	300.15	1.00426	7.32	310.15	1.00518	9.42
291.15	1.00364	3.27	301.15	1.00398	7.59	311.15	1.00481	9.42
292.15	1.00344	3.68	302.15	1.00368	7.79	312.15	1.00442	9.57
293.15	1.00324	3.3	303.15	1.00338	7.54	313.15	1.00404	9.88
313.15			323.15			333.15		
T [K]	ϱ [g·cm^{-3}]	STD [g·cm^{-3}] ·10^5	T [K]	ϱ [g·cm^{-3}]	STD [g·cm^{-3}] ·10^5	T [K]	ϱ [g·cm^{-3}]	STD [g·cm^{-3}] ·10^5
313.15	1.00978	9.46	323.15	1.01332	10.40	333.15	1.01865	5.79
314.15	1.00938	9.09	324.15	1.01284	10.14	334.15	1.01810	5.73
315.15	1.00897	9.09	325.15	1.01236	10.40	335.15	1.01752	4.92
316.15	1.00856	8.73	326.15	1.01186	10.14	336.15	1.01692	5.25
317.15	1.00813	9.09	327.15	1.01137	10.40	337.15	1.01630	7.76
318.15	1.00770	9.09	328.15	1.01086	10.61	338.15	1.01567	10.87
319.15	1.00726	9.09	329.15	1.01035	10.21	339.15	1.01504	14.84
320.15	1.00682	8.73	330.15	1.00983	10.21	340.15	1.01439	18.67
321.15	1.00637	8.73	331.15	1.00931	10.21	341.15	1.01374	22.40
322.15	1.00591	9.09	332.15	1.00878	10.21	342.15	1.01308	26.13
323.15	1.00544	9.09	333.15	1.00825	10.03	343.15	1.01243	28.47
